# Momento difícil para la salud y el bienestar de la niñez

**Published:** 2021-09-22

**Authors:** Ernesto Durán-Strauch

**Affiliations:** 1 Profesor de Pediatría y coordinador del Observatorio sobre Infancia Facultad de Medicina, Universidad Nacional de Colombia Bogotá, D.C., Colombia Universidad Nacional de Colombia Universidad Nacional de Colombia BogotáD.C Colombia

En febrero de 2020 la Comisión OMS-UNICEF-Lancet, creada para analizar la situación de salud de la niñez a nivel global, publicó su informe. En él señalaba:

"[...] A pesar de los extraordinarios progresos lógranos en los últimos decenios en lo concerniente a supervivencia, nutrición y educación, los niños afrontan actualmente un futuro incierto. El cambio climático, la degradación ecológica, las poblaciones migrantes, los conflictos, la desigualdad generalizada y las prácticas comerciales depredadoras [...],

amenazan la salud y el futuro de los niños en todos los países ^1^.

El informe marcó un giro en la agenda de salud y bienestar de la niñez centrada en el control de las enfermedades transmisibles y la desnutrición, y propuso ampliarla para incluir las enfermedades crónicas y otros problemas derivados de las intervenciones del hombre en su entorno natural y social.

El informe se ubica en la "transición epidemiológica" en la salud de la niñez en los países de ingresos altos a finales del siglo pasado y en aquellos de ingresos medios a principios de este siglo, circunstancia que en ese momento predominaba a nivel global. En ese (contexto, se podría hablar de un "mosaico epidemiológico" en el que los problemas relacionados con el saneamiento básico y la pobreza, en disminución a nivel global y propios de los países de menos ingresos, coexisten con aquellos relacionados con la crisis ambiental, la esfera psicosocial y los comportamientos humanos de la situación de salud de la niñez, propios de los países de mayores ingresos.

Un mes después de publicado el informe, la Organización Mundial de la Salud (OMS) declaró la pandemia por COVID-19 y el mundo tomó medidas radicales para disminuir su propagación. Ello produjo un cambio profundo en los perfiles de salud y enfermedad, el cual aún no se ha acabado de caracterizar y entender plenamente. Con este brusco viraje se retornó - de alguna manera- a la primera etapa de la “transición epidemiológica” propuesta por Omran en 1971, quien caracterizó la siguiente etapa como la de “pandemias en retroceso” ^2^.

La sindemia actual ha producido múltiples efectos en la salud y el bienestar de la niñez ^3^. Se incrementaron, por ejemplo, las muertes maternas (hasta el 28 de agosto de 2021 se habían presentado 361 muertes maternas tempranas en Colombia, es decir, un aumento del 35,2 % con respecto al mismo periodo de 2020, y del 87 % con respecto al 2019) ^4^; aumentaron las defunciones neonatales por complicaciones obstétricas (de una tasa de 35,5 muertes por cada 100.000 nacidos vivos en el segundo trimestre de 2019, el país pasó a una tasa de 63,6 en el mismo periodo de 2020) ^5^; se incrementó significativamente la población que vive en la pobreza (aumentó de 35,7 % en el 2019 a 42,5 % en el 2020) y en la pobreza extrema (aumentó de 9,6 % en el 2019 al 15,1 % en el 2020) ^6^; se deterioró la seguridad alimentaria, lo que conlleva aumento de la desnutrición (en el 2019, el 89,3 % de los hogares consumía en promedio tres comidas al día y, en junio de 2021, solo el 66,2 % lo hacía) ^7^, así como del sobrepeso y la obesidad ^8^; aumentaron los conflictos en el interior de las familias, así como los casos reportados de maltrato infantil (9.011 en el 2019 y 13.266 en el 2020, con un aumento del 47 %) ^9^; hubo un deterioro de la salud mental tanto de padres y cuidadores como de los niños y adolescentes ^10^; aumentaron las dificultades para el acceso a la educación virtual (al inicio de la pandemia, el 37,3 % de los hogares colombianos poseía computador de escritorio, portátil o tableta y el 73,8 % de los mayores de 5 años tenía celular) ^11^, así como la deserción escolar ^12^; además, disminuyó el tiempo para el juego, el deporte y las actividades al aire libre ^13^; aumentó la orfandad debido a la muerte de los cuidadores ^14^, y se incrementó la exposición a la pornografía, el ciberacoso y el engaño pederasta *(grooming)*^15^.

Otro efecto de la sindemia ha sido el detrimento en la cantidad y la calidad de un número significativo de actividades de promoción, prevención, diagnóstico, atención y rehabilitación, orientadas a la niñez y la adolescencia. La encuesta Pulso Social del DANE evidenció que, en julio de 2021, el 35,7 % de las personas afirmó que se había visto obligado a faltar a citas médicas o controles de desarrollo integral infantil desde el inicio de la cuarentena ^16^, situación que afecta la detección temprana y la intervención oportuna en la salud de los menores. Entre las actividades que más se han visto afectadas, están los controles integrales de salud, la vacunación, el seguimiento a niños y adolescentes con enfermedades crónicas o en situación de discapacidad, los procesos de rehabilitación, los controles de la gestación, las consultas de planificación familiar y el acceso a métodos anticonceptivos, la interrupción voluntaria del embarazo, la asistencia oportuna a urgencias, los controles de salud oral y salud visual, las consultas por nutrición, y las intervenciones del Sistema Nacional de Bienestar Familiar, entre otras.

Si bien toda la población en edad pediátrica se ha visto afectada, el impacto ha sido mayor en niños y adolescentes en condición de vulnerabilidad, es decir, los más pobres, los desplazados, los migrantes, los indígenas y otras minorías étnicas, así como quienes están en instituciones de protección y en situación de discapacidad, evidenciándose no solo la vulneración de su derecho a la salud, sino de todos sus derechos.

La salud y el bienestar de la niñez y la adolescencia atraviesan, entonces, un complejo momento en el que se combinan el deterioro reciente de las condiciones de vida y de la situación nutricional de un porcentaje importante de la población, con las dificultades en el acceso a una educación de calidad, los demás efectos directos e indirectos de la sindemia, y el proceso de transición que menciona el citado informe ^1^, en el cual aumentan los problemas de salud derivados de las afectaciones ambientales y los hábitos de nutrición y actividad física inadecuados, y persisten las enfermedades transmisibles y los déficits nutricionales.

Hasta principios del 2020, se percibía en los discursos oficiales en Colombia un relativo optimismo en torno a la mejoría de la salud de la niñez con base en la disminución progresiva de la mortalidad infantil, la cobertura sostenida de los índices de vacunación, la disminución del embarazo adolescente y el aumento progresivo de la escolaridad desde la primera infancia, entre otros, aunque persistían problemas críticos como la mortalidad por desnutrición o asociada con esta y los altos índices de maltrato, abuso sexual y muertes violentas de adolescentes, entre otros indicadores no favorables.

Hasta el 28 de agosto de 2021, 18 meses después de iniciada la sindemia, la infección por COVID-19 ha producido 423.442 casos (tasa de 29,6 por 1.000), 8.015 hospitalizaciones (1,9 %), 1.327 ingresos a unidades de cuidados intensivos (0,31 %) y 234 muertes (tasa de 1,6 por 100.000) en menores de 18 años ^17^, y aunque estas son cifras bastante inferiores a las de los demás grupos etarios, los efectos indirectos han sido críticos para las nuevas generaciones.

Entretanto, el sistema de salud colombiano tiene sus energías y recursos centrados en la contención del COVID-19, desconociendo la gravedad de los demás problemas de salud de la niñez, los cuales requieren una pronta intervención porque están en juego la salud, y el bienestar actual y futuro de los más jóvenes.

El sistema de salud ha reaccionado en aspectos importantes como la vacunación, la cual ha tendido a recuperar las coberturas previas a la sindemia, pero esto no es suficiente; necesitamos prontamente *descovidificar* la agenda de salud de la niñez ^18^, retomando las acciones y programas que el país venía adelantando, atendiendo problemas emergentes como las repercusiones en la salud mental y mejorando la cobertura de los diferentes servicios de salud dirigidos a la población menor de 18 años, pero, sobre todo, garantizando un ingreso digno a las familias para que puedan cuidar adecuadamente a sus hijos.

## References

[1] The WHO-UNICEF- Lancet Commissioners (2020). After COVID-19, a future for the world's children?. Lancet.

[2] Omran OR (1971). The epidemiologic transition. The Milbank Memorial Fund Quarterly.

[3] Horton R (2020). COVID-19 is not a pandemic. Lancet.

[4] Instituto Nacional de Salud (2021). Boletín epidemiológico semana 34 de 2021.

[5] Fundación Éxito (2021). Efectos del COVID-19 en la primera infancia de Colombia. No es solo una emergencia.

[6] DANE Pobreza monetaria nacional 2020.

[7] DANE Encuesta Pulso Social, decimosegunda ronda, junio 2021 - julio 2021.

[8] Amézquita M (2021). El impacto de COVID-19 en la obesidad pediátrica. Andes Pediatr.

[9] Alianza por la Niñez Colombiana - Niñez YA (2021). La pandemia tiene en crisis los derechos de la niñez.

[10] Rodríguez-Gama A (2021). La salud mental durante la pandemia por COVID-19. Rev Fac Med.

[11] DANE Boletín Técnico Indicadores básicos de tenencia y uso de tecnologías de la información y las comunicaciones en hogares y personas de 5 y más años de edad.

[12] Seusan LA, Maradiegue R (2020). Educación en pausa. Una generación de niños y niñas en América Latina y el Caribe está perdiendo la escolarización debido al COVID-19.

[13] Villaquirán AF, Ramos OA, Jácome SJ, Meza M (2020). Actividad física y ejercicio en tiempos de COVID-19. Rev CES Med.

[14] Hillis SD, Unwin HJ, Chen Y, Cluver L, Sherr L, Goldman PS (2021). Global minimum estimates of children affected by COVID-19-associated orphanhood and deaths of caregivers: A modelling study. Lancet.

[15] Save the Children (2020). (Des)información sexual: pornografía y adolescencia.

[16] DANE (2021). Encuesta Pulso Social, decimotercera ronda, julio 2021.

[17] (2021). @pvasquezcolpicu (Pablo Vásquez-Hoyos). "#COVID19: Reporte semana 22 a 28 de agosto 2021. Colombia. Pediatría." Twitter.

[18] Durán-Strauch E (2020). Recuperar el tiempo perdido. Pediatría.

